# The Influence of Blood Transfusion Indexed to Patient Blood Volume on 5-Year Mortality After Coronary Artery Bypass Grafting—An EuroSCORE II Adjusted Spline Regression Analysis

**DOI:** 10.3390/jcdd12080287

**Published:** 2025-07-28

**Authors:** Joseph Kletzer, Maximilian Kreibich, Martin Czerny, Tim Berger, Albi Fagu, Laurin Micek, Ulrich Franke, Matthias Eschenhagen, Tau S. Hartikainen, Mirjam Wild, Dalibor Bockelmann

**Affiliations:** 1 Department of Cardiovascular Surgery, University Heart Center Freiburg—Bad Krozingen, University Medical Center Freiburg, 79110 Freiburg, Germany; 2 Faculty of Medicine, University of Freiburg, 79085 Freiburg, Germany; 3 Department of Cardiology and Angiology, University Heart Center Freiburg—Bad Krozingen, University Medical Center Freiburg, 79110 Freiburg, Germany; 4 Staff Unit for Medical Strategy and Cooperation, University Clinic Freiburg, 79106 Freiburg, Germany

**Keywords:** coronary artery bypass grafting, blood transfusion, survival analysis, cardiac surgery, coronary artery disease

## Abstract

**Background:** While timely blood transfusion is critical for restoring oxygen-carrying capacity after coronary artery bypass grafting (CABG), allogeneic blood product transfusions are independently associated with increased long-term mortality, necessitating a risk-stratified approach to balance oxygen delivery against immunological complications and infection risks. **Methods:** We retrospectively analyzed 3376 patients undergoing isolated CABG between 2005 and 2023 at a single tertiary center. Patients who died during their perioperative hospital stay within 30 days were excluded. Transfusion burden was assessed both as the absolute number of blood product units (packed red blood cells, platelet transfusion, fresh frozen plasma) and as a percentage of calculated patient blood volume. The primary outcome was all-cause mortality at 5 years. Flexible Cox regression with penalized smoothing splines, adjusted for EuroSCORE II, was used to model dose–response relationships. **Results:** From our cohort of 3376 patients, a total of 137 patients (4.05%) received >10 units of packed red blood cells (PRBC) perioperatively. These patients were older (median 71 vs. 68 years, *p* < 0.001), more often female (29% vs. 15%, *p* < 0.001), and had higher preoperative risk (EuroSCORE II: 2.53 vs. 1.41, *p* < 0.001). After 5 years, mortality was 42% in the massive transfusion group versus 10% in controls. Spline regression revealed an exponential increase in mortality with transfused units: 14 units yielded a 1.5-fold higher hazard of death (HR 1.46, 95% CI 1.31–1.64), rising to HR 2.71 (95% CI 2.12–3.47) at 30 units. When transfusion was indexed to blood volume, this relationship became linear and more tightly correlated with mortality, with lower maximum hazard ratios and narrower confidence intervals. **Conclusions:** Indexing transfusion burden to the percentage of patient blood volume replaced provides a more accurate and clinically actionable predictor of 5-year mortality after CABG than absolute unit counts. Our findings support a shift toward individualized, volume-based transfusion strategies to optimize patient outcomes and resource stewardship in a time of limited availability of blood products.

## 1. Introduction

Perioperative blood transfusion is commonly used in cardiac surgery, particularly during and after coronary artery bypass grafting (CABG), to manage anemia and maintain hemodynamic stability [1,2,3]. However, higher transfusion volumes have been consistently linked to increased morbidity and mortality in both the short and long term [4,5,6]. Notably, each additional unit of transfused red blood cells independently raises the risk of adverse outcomes, including a two- to three-fold increase in long-term mortality for patients receiving two or more units [4,5]. Despite these risks, transfusion decisions often rely on fixed unit thresholds that may not account for individual patient factors such as physiology, blood volume, and comorbidities [7]. This limitation is particularly relevant as the cardiac surgical population becomes increasingly diverse and complex [8]. Recent recommendations advocate for more individualized, physiology-based transfusion strategies, though evidence is limited on whether indexing transfusion burden to the percentage of patient blood volume replaced improves risk stratification compared with traditional unit-based methods [7,9,10]. Addressing this gap, our study evaluates the relationship between transfusion burden—measured both as absolute units and as a percentage of calculated blood volume—and long-term mortality after CABG, with the aim of informing safer, more precise, and resource-conscious transfusion practices in contemporary cardiac surgery.

## 2. Patients and Methods

### 2.1. Ethical Statement

IRB approval for this study (reference number 23-1389-S1-retro) was granted by the Ethics Committee of the University of Freiburg on 23 November 2023. Due to the retrospective nature of the research, the requirement for obtaining individual informed consent from participants was waived.

### 2.2. Data Collection Instruments

Patient data for those undergoing isolated coronary artery bypass grafting (CABG) were collected using the dedicated retrospective CABG database maintained by the Department of Cardiovascular Surgery at the University Medical Center Freiburg. Survival outcomes were ascertained by manually checking records from the federal resident registration office. Information regarding coronary reinterventions was obtained from institutional medical records and was manually categorized as either surgical or interventional procedures. The study included cases from January 2005 to February 2023. Datapoints where endpoints could not be conclusively ascertained were excluded from the analysis. To focus on the long-term prognostic impact of blood product transfusion, patients who died within 30 days of surgery were excluded to remove those succumbing to immediate postoperative complications. This approach aims to assess the association between transfusion and subsequent mortality, independent of early deaths directly related to surgical events. Of the initial 3657 patients who underwent CABG during the study period, a total of 3376 were included in the final analysis after excluding those with missing data on mortality or number of perioperative blood products. These were then divided into two groups based on the overall number of PRBCs transfused. There is no universally accepted definition for massive transfusion in the context of cardiac surgery; for the purposes of this study, we defined the massive transfusion subgroup as patients who received more than 10 units of PRBCs during their perioperative stay [11].

### 2.3. Definition of Primary Outcome Measure and Model Parameters

As the primary outcome measure, we chose all-cause mortality after 5 years. To calculate the volume of the blood products transfused, we used information provided by our local institute for transfusion medicine and gene therapy. During the timeframe of our study, the average volume of a single unit of blood product was 282 mL for PRBCs, 235 mL for platelet transfusion (PT), and 311 mL for fresh frozen plasma (FFP). We then multiplied these volumes with the number of units transfused to arrive at the total volume transfused. Patient blood volume was then calculated using the formula:

$$Blood\;Volume\;(mL)=\left\{\begin{array}{c} Weight\left(kg\right)\times 70\;if\;Sex=Male\\ Weight\left(kg\right)\times 61\;if\;Sex=Female \end{array}\right\}$$

We selected this formula for its simplicity and ease of application in clinical practice. While the volume factor for male patients is commonly accepted to be approximately 70 mL/kg, the appropriate factor for female patients is less clearly established, with most sources recommending a value between 60 and 65 mL/kg, depending on the body mass index (BMI) of the patient [12,13]. It is generally estimated that, with additional adipose tissue, this coefficient shrinks and that a coefficient of 65 mL/kg is usually only found in normal weight individuals. Therefore, we opted for around 61 mL/kg, as the recorded BMI of our cohort was above 25 kg/m^2^, and therefore, generally more adipose tissue could be assumed [14,15]. Additionally, as per our local protocol, all patients usually receive acetylsalicylic acid after CABG surgery. P2Y12 inhibitors are added for 12 months in the context of myocardial infarction. If any indication for anticoagulation such as atrial fibrillation arises, acetylsalicylic acid is discontinued as long as therapeutic anticoagulation is given.

### 2.4. Statistical Analysis

Statistical analyses were conducted using R (version 4.3.3) within the RStudio environment. Data visualization was performed with ggplot2 (version 3.5.0), an R package designed for creating high-quality graphical representations of data.

The normality of data distributions was evaluated using the Shapiro–Wilk test, which assesses whether a sample is drawn from a normally distributed population. For data that followed a normal distribution, results were reported as mean ± standard deviation and comparisons between groups were made using the unpaired t-test. For data that did not meet normality assumptions, results were presented as median with interquartile range, and group differences were assessed using the Mann–Whitney U test. Categorical variables were compared using the chi-squared test. The comparison and differentiation of massive transfusion was done to illustrate the characteristics of patients who received more blood products. However, it was not used at any point during modeling.

A flexible Cox proportional hazards model was implemented to examine the dose–response relationship between blood product unit transfusion quantities and 5-year mortality risk. The model incorporated a penalized smoothing spline (P-spline) for unit counts using 1.9 effective degrees of freedom, allowing for nonlinear pattern detection while preventing overfitting. The analysis adjusted for baseline surgical risk using EuroSCORE II as a covariate. Model selection for the spline regression analyses was guided by comparing candidate models with varying degrees of freedom and knot placements. We calculated the Akaike Information Criterion (AIC) and the Bayesian Information Criterion (BIC) for each model specification, selecting the model with the lowest criterion value as optimal. As such, some covariates such as preoperative hemoglobin were omitted from the final model.

Hazard ratios were centered at 2 units (reference value) for clinical interpretability, as this was the median number of transfused units in our cohort. The nonlinear association was visualized through a logarithmic-scale plot of hazard ratios against transfusion quantities, featuring a reference line at HR = 1 and shaded 95% confidence bands.

Quantile-specific hazard ratios were calculated at the 25th, 50th, 75th, and 100th percentiles of unit numbers and blood product transfusion volumes. Statistical significance for these estimates was determined using Wald tests derived from the ratio of log hazard ratios to their standard errors. All analyses were performed in R (version 4.3.3) using the RStudio environment (Posit team (2024). RStudio: Integrated Development Environment for R. Posit Software, PBC, Boston, MA, USA. URL http://www.posit.co/) and the survival (version 3.8.3), with graphical representations created via ggplot2 (version 3.5.0.) from those addressed by the spline transformation. No significant violations of model assumptions were detected, supporting the appropriateness of the applied analytical approach.

## 3. Results

### 3.1. Patients’ Characteristics

In the overall patient cohort, a total of 137 (4.05%) patients underwent massive transfusion as defined previously. Forty (29%) of those were female, while only 15% were female in the subgroup which did not receive massive transfusion (*p* < 0.001). Patients who underwent massive transfusion were also older [71 (66–76) years vs. 68 (61–74); *p* < 0.001] and had a higher preoperative risk of 30-day mortality [EuroSCORE II: 2.53 (1.62–5.16) vs. 1.41 (0.95–2.22); *p* < 0.001]. Such findings are also mirrored in the significantly higher rate of prior myocardial infarction [77 (56%) vs. 1055 (33%); *p* < 0.001], prior stroke [19 (14%) vs. 182 (5.6%); *p* < 0.001], and other comorbidities. A more detailed account of the patient baseline characteristics are shown in Table 1.

### 3.2. Intraoperative Data

There was no significant difference in the number of bypasses performed (*p* = 0.300), nor was there any significant difference in percentage of complete revascularization between the two investigated groups (*p* = 0.200). Patients with perioperative massive transfusion were subjected to a significantly longer time on cardiopulmonary bypass [103 (87–134) min vs. 89 (74–106) min; *p* < 0.001] and to an overall longer duration of surgery [240 (210–290) min vs. 220 (190–250) min; *p* < 0.001]. However, at the same time, the number of performed bypasses did not differ [3.02 (±0.95) vs. 3.13 (±0.87); *p* = 0.300]. The intraoperative results are summarized in Table 2.

### 3.3. Clinical Outcomes and Safety Endpoints

After 5 years, 58 (42%) patients who had received massive transfusion died, while only 323 (10%) reached the same outcome in the control group. The rate of coronary reintervention was also much higher in the massive transfusion group, with 29 (22%) patients having to undergo either repeat surgical or catheter revascularization after 5 years compared with 242 (7.6%) in the control group. Of the endpoints investigated, all except for postoperative permanent pacemaker implantation (*p* = 0.130) reached significance. Table 3 illustrates the postoperative outcomes in our cohort.

### 3.4. Spline Regression

Overall, the EuroSCORE II adjusted splines showed a substantial increase in the hazard of death with each transfused blood product.

**Figure 1:**
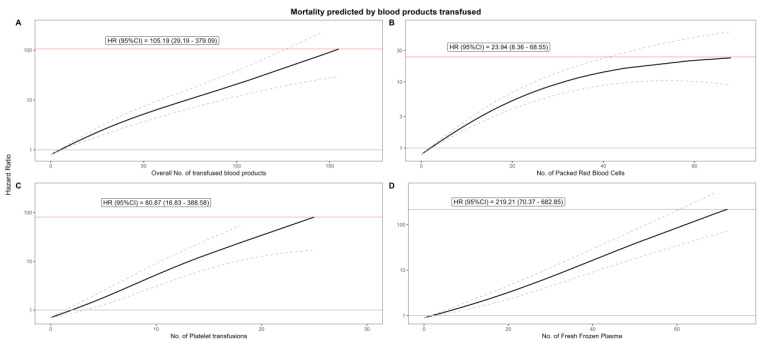
Smoothed P-spline models display the relationship between the number of blood product units transfused and 5-year mortality. The y-axis (on the left of each panel) shows the hazard ratio (HR) for mortality, while the x-axis represents the number of transfused units for each blood product. The solid black line indicates the estimated hazard ratio, with dashed lines illustrating the 95% confidence intervals. The red horizontal line and label highlight the maximum observed hazard. Panel (**A**): overall units transfused; Panel (**B**): packed red blood cells transfused; Panel (**C**): platelet transfusions transfused; Panel (**D**): fresh frozen plasma transfused; HR: hazard ratio; and No.: number of units transfused. Confidence intervals are shown as dashed lines, and the y-axis is labeled on the left for all panels.

**Effect of units of blood product**: There was an exponential increase in mortality with each unit of PRBCs, platelets, or FFP transfused. Transfusion of 14 (1st quartile) blood products in total lead to an almost 1.5-fold increase [HR 1.462 (1.305–1.637); *p* < 0.001] in the hazard of death after 5 years compared with only receiving 2 (reference) units of any kind. After 30 units, this increased to an HR of 2.711 (2.116–3.474). An exponential increase in the HR was observed with each transfused unit of platelets or FFP, whereas with PRBC transfusions, the HR reached a clear plateau beginning at around 40 units. For PRBCs, the hazard started to peak at around the 3rd quartile [32 units: 10.415 (6.997–15.501); *p* < 0.001] and reached its maximum at the 4th quartile [71 units: 23.944 (8.364–68.546); *p* < 0.001]. Table 4 and Figure 1 depict the hazard of death for each quartile of transfused blood products, as calculated in our spline model.

**Figure 2:**
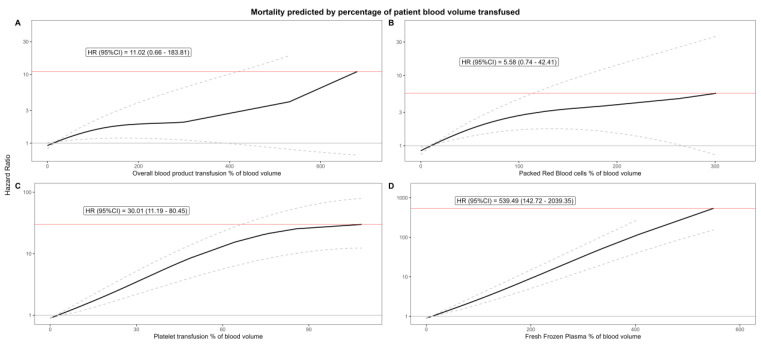
Smoothed P-spline models display the relationship between the % of blood volume transfused and 5-year mortality. The y-axis (on the left of each panel) shows the hazard ratio (HR) for mortality, while the x-axis represents the number of transfused units for each blood product. The solid black line indicates the estimated hazard ratio, with dashed lines illustrating the 95% confidence intervals. The red horizontal line and label highlight the maximum observed hazard. Panel (**A**): overall % transfused; Panel (**B**): packed red blood cells transfused; Panel (**C**): platelet transfusions transfused; Panel (**D**): fresh frozen plasma transfused; HR: hazard ratio; and No.: number of units transfused. Confidence intervals are shown as dashed lines, and the y-axis is labeled on the left for all panels.

**Effect of percentage of patient blood volume transfused**: The previously evident exponential increase is severely dampened. Additionally, while previously plateauing, the effect of PRBCs now shows an almost linear character. Previously, the model clearly showed an exponential relationship (*p* < 0.001), which now only barely clears statistical significance (*p* = 0.012). The models for the other types of blood products also show a dampened increase, with lower maximum hazard ratios and tighter confidence intervals. A detailed account of the results are shown in Table 4 and Figure 2.

## 4. Discussion

This study’s three main findings can be summarized as follows: (**I**) When excluding immediate in-hospital mortality, the number of transfused blood product units exhibits an exponential relationship with 5-year mortality, underscoring the cumulative hazard prediction of high-volume transfusion. (**II**) However, when transfusion burden is indexed to the percentage of a patient’s innate blood volume replaced, this relationship linearizes, demonstrating a more physiologically grounded and clinically actionable correlation with mortality. (**III**) These results collectively advocate for resuscitation strategies that prioritize percentage-based thresholds over fixed unit counts, a paradigm shift that could harmonize transfusion safety with precision medicine, particularly in populations with extreme body habitus or comorbidities affecting blood volume.

**Baseline characteristics**: In our cohort, patients undergoing massive transfusion constituted 4.05% of the total, with a notably higher proportion of females, older age, and greater preoperative risk compared with those not requiring massive transfusion. These patients also exhibited a significantly higher burden of comorbidities, including prior myocardial infarction and stroke, and experienced longer cardiopulmonary bypass and operative times, although the number of bypasses performed was similar between groups. The observed demographic and clinical profiles align with prior studies in cardiac surgery, which consistently report that patients requiring massive transfusion are older and have higher baseline risk scores and more comorbidities. These patients are more likely to require greater volumes of blood products because they often have reduced physiological reserves and are at higher risk for both bleeding and complications during complex cardiac surgery [16,17,18,19]. These findings also reflect the larger proportion of off-pump cases in the less transfused subgroup. Recent large cohort studies have confirmed that off-pump CABG is associated with substantially lower red blood cell transfusion requirements compared with on-pump procedures, which is thought to contribute to improved long-term outcomes in these patients. This difference is primarily attributed to the avoidance of using a cardiopulmonary bypass pump, resulting in less hemodilution and coagulopathy and thereby reducing the need for transfusion [20,21]. Our findings of increased 5-year mortality and higher rates of coronary reintervention in the massive transfusion group are also consistent with the literature, which demonstrates a clear association between transfusion volume and adverse outcomes, including stepwise increases in mortality with higher transfusion thresholds [5,17,18]. These results underscore the importance of early identification and risk stratification for patients likely to require massive transfusion, as well as the need for tailored perioperative management protocols to mitigate the heightened risk of morbidity and mortality in this vulnerable subgroup.

**Number of blood products predicts mortality:** Our analysis demonstrated an exponential relationship between the number of transfused blood product units and 5-year mortality, with hazard ratios escalating with each additional unit and PRBC-specific risks plateauing beyond 40 units. These findings align with multicenter studies showing mortality rising from 6.0% at 10 units to 38.9% at 40 units of PRBCs within 24 h [18], consistent with dose-dependent toxicity patterns observed in trauma and cardiac surgery populations [22,23,24]. Mechanistically, this nonlinear risk trajectory reflects cumulative transfusion-related immunomodulation (TRIM), endothelial glycocalyx shedding from free hemoglobin, and iron-mediated oxidative stress, which all synergistically drive multi-organ dysfunction [22]. The PRBC-specific hazard plateau at extreme volumes may signal adaptive responses, such as macrophage-mediated iron sequestration or coagulopathy exacerbation, as observed in trauma resuscitation studies where PRBC-dominated protocols lose efficacy beyond physiological thresholds [18,22,25]. Platelet and fresh frozen plasma (FFP) transfusions may each contribute to adverse outcomes through distinct mechanisms. Platelet transfusions have been shown to promote a pro-inflammatory and pro-aggregatory state, increasing levels of mediators such as P-selectin and CD40 ligand, and can induce TRIM independently of white blood cells, potentially heightening the risk of postoperative complications and thrombotic events [26,27]. FFP transfusion, while used to manage coagulopathy, is associated with an increased risk of acute lung injury, circulatory overload, and higher short-term mortality in cardiac surgery patients and may not reduce bleeding or improve long-term outcomes [28]. These findings underscore the importance of judicious use of platelet and FFP transfusions and the need for individualized transfusion strategies in cardiac surgery.

Despite adjustments for EuroSCORE II and exclusion of in-hospital deaths, residual confounding persists from unmeasured variables such as frailty. However, transfusion burden remains a potent risk stratifier after hospital discharge, as evidenced by national health and nutrition examination survey (NHANES) data showing 84% higher all-cause mortality (HR 1.84) over 10.8 years in transfused patients [16] and by myocardial infarction studies reporting doubled 5-year mortality in transfused versus non-transfused cohorts [29]. Clinically, these data have sparked a controversial debate around optimization of the transfusion strategy in different clinical scenarios [30,31], earlier transition to factor concentrates in refractory hemorrhage, and surveillance after hospital discharge for iron overload in patients receiving >10 units [22,24]. While confounding complicates causal interpretation, transfusion volume serves as a clinically actionable biomarker of cumulative physiological stress, offering granular mortality prediction unmatched by traditional risk scores [16,18,29].

**Association of percentage of blood volume transfused:** When transfusion burden was indexed to the percentage of a patient’s innate blood volume replaced, the previously observed exponential increase in mortality risk with transfused units was markedly attenuated, yielding a nearly linear association for PRBCs and tighter confidence intervals overall. This shift toward linearity is consistent with large-scale analyses demonstrating that mortality risk from transfusion is more physiologically grounded when considered relative to patient size and circulating volume rather than absolute unit counts [32]. For example, studies in both trauma and burn populations have shown that risk curves for transfusion-related mortality become less steep and more predictable when adjusted for body weight or blood volume, supporting the clinical relevance of individualized thresholds [25,33]. Mechanistically, this approach accounts for the fact that a given number of transfused units represents a vastly different physiological insult depending on patient blood volume, thereby reducing confounding from body habitus and improving the accuracy of risk stratification. These findings suggest that indexing transfusion burden to blood volume offers a more actionable and equitable framework for predicting outcomes and guiding transfusion strategies, potentially harmonizing practice across diverse patient populations and reducing the risk of both over- and under-transfusion [25,32,33].

**Percentage transfused is better suited for risk prediction:** The shift toward percentage-based transfusion thresholds, as highlighted by our findings, not only enhances clinical precision but also offers substantial benefits in resource management and cost containment. Blood transfusions represent a significant financial burden, with studies reporting unit costs ranging from $219 to $634 for PRBCs and up to $409 for FFP, when accounting for procurement, storage, testing, and labor [34,35]. Inappropriate or inadequately indicated transfusions further amplify this impact, with annual costs of unnecessary blood product use in some settings reaching up to $34 million and single institutions incurring hundreds of thousands of dollars in avoidable expenses each year [36]. Our results suggest that percentage-based thresholds can more accurately match transfusion needs to individual physiological requirements, reducing both over- and under-transfusion, particularly in patients at the extremes of body habitus or with comorbidities affecting blood volume. This approach aligns with evidence from cost-effectiveness analyses and protocol-driven interventions, which have demonstrated that restrictive and individualized transfusion strategies can lead to significant reductions in both transfusion rates and overall costs, with savings of up to 56% reported following protocol implementation [37,38]. Mechanistically, by tailoring transfusion to the patient’s actual circulatory volume rather than relying on fixed unit counts, hospitals can better steward their blood supply, minimize wastage, and ensure that this costly and finite resource is directed where it is most beneficial. Ultimately, adopting percentage-based transfusion thresholds represents a paradigm shift that harmonizes patient safety, clinical efficacy, and economic stewardship in transfusion medicine [34,35,36,37,38].

Overall, these findings highlight the importance of implementing patient blood management (PBM) programs that use evidence-based multidisciplinary strategies, such as preoperative anemia correction and restrictive transfusion thresholds, to optimize outcomes and preserve blood resources. Adoption of PBM protocols in cardiac surgery has been shown to reduce transfusion rates and improve recovery in patients at high risk for transfusion [39,40].

## 5. Limitations

Firstly, despite a rigorous adjustment for EuroSCORE II and the use of P-splines, residual confounding from unmeasured variables (e.g., genetic predispositions, frailty biomarkers) may persist. While our Cox regression with P-splines robustly modeled nonlinear hazard trajectories, residual confounding may persist due to unmeasured genetic factors (e.g., *HFE* mutations affecting iron metabolism) and frailty biomarkers (e.g., gait speed, grip strength), which were unavailable in our dataset. Moreover, our study did not stratify analyses by time period, and evolving transfusion practices over the study duration may have influenced both transfusion patterns and clinical outcomes. Advanced methods like high-dimensional propensity score adjustment or machine learning-based confounder selection were precluded by our single-center cohort’s limited sample size, which risks overfitting in ML models, and the absence of granular omics or physiological waveform data required for such approaches.

Secondly, the 10-unit massive transfusion threshold, while clinically intuitive, lacks consensus definitional support; however, as our study was not designed to compare massive transfusion versus no massive transfusion. The investigated effect using a spline model and the definition of massive transfusion are only used in Table 1, Table 2 and Table 3 for illustrative purposes.

Additionally, this study’s single-center design ensures consistent data collection and management but limits the generalizability of our findings. The patient population may not be representative of broader cardiac surgery cohorts, as demographic characteristics and institutional transfusion protocols can vary significantly between centers. Variability in perioperative transfusion practices, such as thresholds for transfusion, blood conservation strategies, and institutional guidelines, may impact the external applicability of our results. Future multicenter studies are warranted to validate these findings across diverse populations and practice settings.

This study’s strengths include its large sample size (n = 3376) spanning 18 years, granular transfusion volume data, and innovative application of spline regression to model nonlinear mortality risk—a methodological advance over traditional categorical analyses. The blood volume-indexed approach addresses a critical gap in transfusion research by reconciling disparate body habitus effects—a limitation plaguing prior unit-based studies. By excluding in-hospital deaths, we isolated 5-year transfusion toxicity from perioperative survivorship bias—a design rigor absent in many observational analyses.

## 6. Conclusions

Indexing transfusion burden to the percentage of patient blood volume replaced offers a more accurate and clinically relevant predictor of 5-year mortality after coronary artery bypass grafting than absolute unit counts. This individualized, volume-based approach supports a paradigm shift in transfusion practice, promising to enhance patient safety, improve risk stratification, and promote more efficient use of blood resources in perioperative care.

## Figures and Tables

**Table 1:** Patient baseline characteristics; BMI: body mass index; COPD: chronic obstructive pulmonary disease; NYHA: New York Heart Association (Score); LVEF: left ventricular ejection fraction.

		Extent of Perioperative Transfusion		
Characteristic	Overall, N = 3376 ^1^	Massive Transfusion, N = 137 ^1^	No Massive Transfusion, N = 3239 ^1^	*p*-Value ^2^
Sex (female)	522 (15%)	40 (29%)	482 (15%)	<0.001
Age (years)	68.00 (62.00, 74.00)	71.00 (66.00, 76.00)	68.00 (61.00, 74.00)	<0.001
BMI (kg/m^2^)	27.40 (25.20, 30.10)	27.60 (24.60, 30.15)	27.40 (25.20, 30.10)	0.8
Diabetes Mellitus	1141 (34%)	58 (42%)	1083 (33%)	0.032
Dyslipidemia	2696 (80%)	114 (83%)	2582 (80%)	0.4
History of hypertension	2816 (84%)	129 (94%)	2687 (83%)	<0.001
History of arrhythmia	283 (8.4%)	23 (17%)	260 (8.0%)	<0.001
History of smoking	1148 (37%)	51 (40%)	1097 (37%)	0.4
Peripheral artery disease	436 (15%)	26 (24%)	410 (14%)	0.007
History of COPD	339 (10%)	20 (15%)	319 (9.9%)	0.072
History of myocardial infarction	1132 (34%)	77 (56%)	1055 (33%)	<0.001
History of stroke	201 (6.0%)	19 (14%)	182 (5.6%)	<0.001
History of malignancy	60 (1.9%)	1 (0.8%)	59 (2.0%)	0.5
NYHA				<0.001
Class 1	339 (11%)	7 (5.4%)	332 (11%)	
Class 2	1437 (47%)	38 (29%)	1399 (48%)	
Class 3	1188 (39%)	74 (57%)	1114 (38%)	
Class 4	79 (2.6%)	11 (8.5%)	68 (2.3%)	
Chronic renal failure	91 (2.8%)	12 (8.8%)	79 (2.5%)	<0.001
EuroSCORE II	1.44 (0.96, 2.28)	2.53 (1.62, 5.16)	1.41 (0.95, 2.22)	<0.001
Preoperative creatinine (mg/dL)	0.95 (0.80, 1.10)	1.00 (0.84, 1.40)	0.95 (0.80, 1.10)	0.017
Preoperative hemoglobin (g/L)	14.30 (13.20, 15.20)	13.60 (12.20, 14.90)	14.40 (13.30, 15.20)	<0.001
Preoperative thrombocyte count (Tsd.)	221.00 (187.00, 262.00)	219.00 (187.00, 264.00)	221.00 (187.00, 262.00)	>0.9
Preoperative CK-MB (IU/L)	14.00 (11.00, 19.00)	14.00 (11.00, 21.00)	14.00 (11.00, 19.00)	0.5
Preoperative hs-cTnT (ng/L)	0.01 (0.01, 0.04)	0.02 (0.01, 0.14)	0.01 (0.01, 0.04)	<0.001
Preoperative LVEF < 30%	86 (2.7%)	11 (8.7%)	75 (2.5%)	<0.001

^1^ n (%); Median (IQR); ^2^ Pearson’s chi-squared test; Wilcoxon rank sum test; Fisher’s exact test.

**Table 2:** Intraoperative characteristics.

		Extent of Perioperative Transfusions		
Characteristic	Overall, N = 3376 ^1^	Massive Transfusion, N = 137 ^1^	No Massive Transfusion, N = 3239 ^1^	*p*-Value ^2^
No. of bypasses	3.12 (0.88)	3.02 (0.95)	3.13 (0.87)	0.3
Complete revascularization	2754 (91%)	113 (88%)	2641 (91%)	0.2
No. of affected vessels				0.001
1 vessel	25 (1%)	6 (4%)	19 (1%)	
2 vessels	277 (8%)	8 (6%)	269 (8%)	
3 vessels	3060 (91%)	121 (90%)	2939 (91%)	
Time of cardiopulmonary bypass (min)	90 (74, 107)	103 (87, 134)	89 (74, 106)	<0.001
Time of aortic cross-clamping (min)	69 (55, 83)	75 (60, 92)	68 (55, 83)	0.003
Total time of surgery (min)	220 (190, 250)	240 (210, 290)	220 (190, 250)	<0.001
Off-Pump cases	304 (9.0%)	10 (7.3%)	294 (9.1%)	0.5

^1^ Mean (SD); n (%); Median (IQR); ^2^ Wilcoxon rank sum test; Pearson’s chi-squared test; Fisher’s exact test.

**Table 3:** Postoperative outcomes; LVEF: left ventricular ejection fraction.

		Extent of Perioperative Transfusions		
Characteristic	Overall, N = 3376 ^1^	Massive Transfusion, N = 137 ^1^	No Massive Transfusion, N = 3239 ^1^	*p*-Value ^2^
Follow-up (years)	6.63 (4.06, 10.62)	4.87 (2.93, 7.77)	6.73 (4.11, 10.73)	<0.001
3-year mortality	241 (7.1%)	41 (30%)	200 (6.2%)	<0.001
5-year mortality	381 (11%)	58 (42%)	323 (10.0%)	<0.001
Coronary reinterventions after 5 years	271 (8.1%)	29 (22%)	242 (7.6%)	<0.001
Coronary reinterventions after 3 years	225 (6.8%)	26 (20%)	199 (6.2%)	<0.001
Coronary reinterventions after 30 days	92 (2.8%)	21 (16%)	71 (2.2%)	<0.001
Surgical coronary reintervention after 5 years	6 (0.2%)	2 (1.5%)	4 (0.1%)	0.022
Surgical coronary reintervention after 3 years	6 (0.2%)	2 (1.5%)	4 (0.1%)	0.022
Surgical coronary reintervention after 30 days	5 (0.1%)	2 (1.5%)	3 (<0.1%)	0.015
Percutaneous coronary reintervention after 5 years	271 (8.1%)	29 (22%)	242 (7.5%)	<0.001
Percutaneous coronary reintervention after 3 years	225 (6.7%)	26 (20%)	199 (6.2%)	<0.001
Percutaneous coronary reintervention after 30 days	92 (2.7%)	21 (16%)	71 (2.2%)	<0.001
Postoperative coronary angiography	602 (18%)	39 (28%)	563 (17%)	<0.001
Length of hospital stay (days)	14.00 (11.00, 17.00)	29.00 (18.00, 39.00)	14.00 (11.00, 17.00)	<0.001
Length of ICU stay (days)	0.96 (0.87, 1.87)	7.13 (2.89, 17.60)	0.95 (0.87, 1.79)	<0.001
No. of erythrocyte transfusions	2.64 (4.43)	18.03 (11.32)	1.99 (2.16)	<0.001
No. of thrombocyte transfusions	0.33 (1.15)	3.00 (4.09)	0.21 (0.60)	<0.001
No. of FFP units	1.90 (4.01)	12.21 (12.51)	1.46 (2.34)	<0.001
% of calculated blood volume transfused	13.40 (0.00, 34.34)	134.17 (96.69, 210.28)	12.01 (0.00, 30.93)	<0.001
% of calculated blood volume transfused by packed red blood cells	8.66 (0.00, 18.96)	74.78 (56.81, 104.85)	7.67 (0.00, 17.27)	<0.001
% of calculated blood volume transfused by platelet transfusion	0.00 (0.00, 0.00)	8.19 (0.00, 16.79)	0.00 (0.00, 0.00)	<0.001
% of calculated blood volume transfused by fresh frozen plasma	0.00 (0.00, 17.77)	49.98 (27.31, 93.92)	0.00 (0.00, 16.46)	<0.001
Postoperative permanent pacemaker	49 (1.5%)	4 (2.9%)	45 (1.4%)	0.13
Postoperative dialysis	100 (3.0%)	45 (33%)	55 (1.7%)	<0.001
Postoperative stroke	67 (2.0%)	13 (9.6%)	54 (1.7%)	<0.001
Postoperative atrial fibrillation	639 (19%)	41 (30%)	598 (19%)	<0.001
Postoperative LVEF < 30%	60 (2.0%)	6 (5.8%)	54 (1.8%)	0.015

^1^ Median (IQR); n (%); Mean (SD); ^2^ Wilcoxon rank sum test; Fisher’s exact test; Pearson’s chi-squared test.

**Table 4:** Results of P-spline regression for 5-year mortality at each quartile for number of units transfused as well as % of total blood volume replaced; Q_n_: Quartile number; CI: Confidence interval.

Q_n_	No. of Units	Hazard Ratio	Lower CI	Upper CI	*p*-Value
**Overall**					
**Q1**	14	1.462131	1.305324	1.637774	<0.001
**Q2**	30	2.711864	2.116761	3.474271	<0.001
**Q3**	50	5.208721	3.659172	7.414458	<0.001
**Q4**	155	105.1939	29.1904	379.0892	<0.001
**Packed Red Blood Cells**					
**Q1**	9	2.062345	1.78387	2.384292	<0.001
**Q2**	19	4.855953	3.676097	6.414487	<0.001
**Q3**	32	10.41509	6.997874	15.50102	<0.001
**Q4**	71	23.94493	8.364491	68.54688	<0.001
**Platelets**					
**Q1**	2	1	0.859157	1.163932	1
**Q2**	6	2.209499	1.506778	3.23995	<0.001
**Q3**	10	5.263469	3.061322	9.049721	<0.001
**Q4**	25	80.87303	16.83177	388.5774	<0.001
**Fresh Frozen Plasma**					
**Q1**	7	1.347946	1.182089	1.537074	<0.001
**Q2**	16	2.422458	1.819668	3.224931	<0.001
**Q3**	30	7.06773	4.435473	11.26211	<0.001
**Q4**	72	219.213	70.37322	682.8498	<0.001
	**% of Total Blood Volume**	**Hazard Ratio**	**Lower CI**	**Upper CI**	***p*** **-Value**
**% Overall**					
**Q1**	17.02213	1.029111	1.004066	1.054781	0.022
**Q2**	32.35479	1.126894	1.066384	1.190837	<0.001
**Q3**	55.26984	1.274282	1.105927	1.468267	<0.001
**Q4**	680.2105	11.01697	0.660317	183.8111	0.094
**% Packed Red Blood Cells**					
**Q1**	11.84874	1.02585	1.000567	1.051771	0.045
**Q2**	21.58163	1.187205	1.1099	1.269894	<0.001
**Q3**	38.07136	1.491357	1.253226	1.774736	<0.001
**Q4**	301.0827	5.584695	0.735416	42.40976	0.096
**% Platelets**					
**Q1**	4.900938	1.097342	1.016071	1.185115	0.017
**Q2**	7.89916	1.246535	1.093389	1.421132	<0.001
**Q3**	13.28434	1.576469	1.266585	1.962171	<0.001
**Q4**	110.0703	30.01043	11.19416	80.45498	<0.001
**% Fresh Frozen Plasma**					
**Q1**	16.87161	1.072355	1.038509	1.107305	<0.001
**Q2**	27.80924	1.204566	1.119126	1.29653	<0.001
**Q3**	46.15955	1.467765	1.278395	1.685186	<0.001
**Q4**	549.667	539.4914	142.7177	2039.347	<0.001

## Data Availability

Due to restrictions by our local ethics committee, the raw data used in this manuscript cannot be shared.

## Abbreviations

PRBCs	packed red blood cells
PT	platelet transfusion
FFP	fresh frozen plasma
